# COVID-19 Associated Imported *Plasmodium vivax* Malaria Relapse: First Reported Case and Literature Review

**DOI:** 10.2147/RRTM.S292157

**Published:** 2021-05-10

**Authors:** Zubair Shahid, Nadia Karim, Fakhar Shahid, Zohaib Yousaf

**Affiliations:** 1 Internal Medicine, Hamad Medical Corporation, Doha, Qatar; 2 General Surgery, Hamad Medical Corporation, Doha, Qatar; 3 Dresden International University, Dresden, Germany

**Keywords:** SARS-CoV-2, fever, headaches, reactivation, co-infection

## Abstract

*Plasmodium vivax* (*P. vivax*) is a protozoan parasite that causes vivax malaria. Disease relapse post-treatment is reported in *P. vivax* co-infection with other bacterial and parasitic infections, but *Plasmodium vivax* reactivation is not very common with viral infections. Early recognition and diagnosis of a *Plasmodium vivax* malaria relapse in a non-endemic region pose a diagnostic dilemma. COVID-19 co-infection compounds this dilemma due to overlapping symptoms. Early diagnosis and treatment are essential for a favorable clinical outcome. We report a middle-aged gentleman with high-grade fever and headaches who had COVID-19 and was found to have a relapse of *Plasmodium vivax* malaria.

## Introduction

Novel coronavirus 2019 has created the COVID-19 pandemic with a spectrum of illness from asymptomatic to multiorgan dysfunction and death. Although the predominant symptoms of COVID-19 are respiratory, causing fever, flu-like symptoms, cough, shortness of breath, it can virtually affect any organ system and can have atypical presentations.^1–4^

*Plasmodium vivax* is one of the leading causes of febrile illness in endemic areas of Asia, Central America, South America, and Africa. Malaria relapses characterize *P. vivax* infections due to dormant parasite in the liver known as hypnozoites.^5^ These hypnozoites can be activated by a systemic illness or, in some cases, by other infectious diseases like salmonella typhi.^6^ We report a case of *P. vivax* malaria relapse associated with a COVID-19 (viral illness) co-infection, suggesting a possible role of COVID-19 in inducing current malarial relapse.

## Case Presentation

A 55-year-old Indian gentleman with a medical history of type 2 diabetes mellitus presented with 5 days of dry cough, high-grade fever, chills, rigors, profuse sweating, and lethargy. There was no chest pain, palpitations, hemoptysis, rash, nausea, vomiting, diarrhea, or alteration in the consciousness level. He reported a history of *P. vivax* malaria in India 1 year back, treated successfully with artemether and lumefantrine, followed by primaquine. There was no history of recent travel, insect bites, contact with animals, or blood transfusions. Relevant history and review of systems were unremarkable.

He was febrile (39.9 degree Celsius), tachycardiac (136 beats per minute), but had no tachypnea (18 breaths per minute), a normal blood pressure (120/80 mm Hg) and was maintaining oxygen saturation of 97% on room air. Examination revealed pharyngeal and tonsillar erythema without any exudates. There was no lymphadenopathy. The chest, abdominal and neurological examinations were within normal limits. Meningeal signs were absent.

Laboratory workup revealed neutrophilic leukocytosis (Table 1). The chest X-ray was within normal limits. Considering the febrile illness and high-pretest probability for COVID-19 based on recent exposure, a nasopharyngeal swab was sent for Genexpert reverse transcriptase-polymerase chain reaction (RT-PCR) for COVID-19 and was found to be positive. A working diagnosis of mild COVID-19 upper respiratory tract infection was made. The patient was started on treatment based on the local COVID-19 management protocol at the time.

**Table 1:** Laboratory Results

Laboratory Tests	Value	Reference Range
White cell count	14.8 ×10^3^/µL	4 ×10^3^/µL–10 × 10^3^/µL
Absolute neutrophil count	12.1 ×10^3^/µL	2 ×10^3^/µL–7 × 10^3^/µL
CRP	88.4 mg/L	<5 mg/L
Platelets	52 × 10^3^/µL	150 ×10^3^/µL–400 ×10^3^/µL
Hematocrit	37.0%	40–50%
BUN	3.4 mmol/L	2.8–8.1 mmol/L
Creatinine	83 µmol/L	62–106 µmol/L
ALT	19 U/L	<40 U/L
AST	18 U/L	<40 U/L
Total bilirubin	28 µmol/L	<21 µmol/L

**Figure 1:**
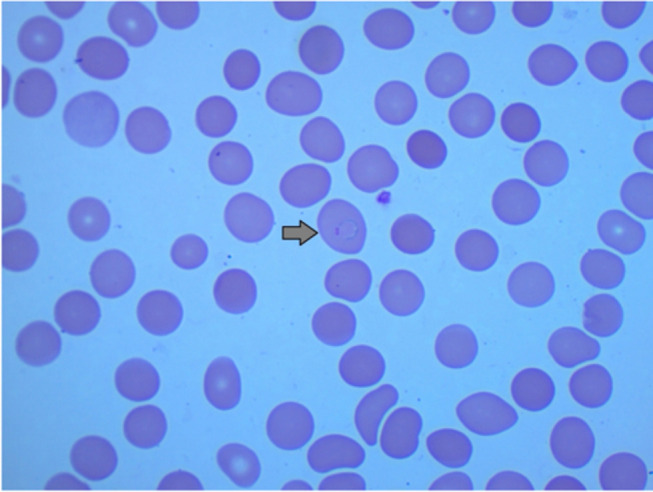
Thin malaria film showing ring-form trophozoite of *P. vivax*.

After treatment for 3 days, there was resolution of cough and pharyngeal erythema, however, on the 4th day patient started to spike high-grade fever of 39.1 degree Celsius. Sepsis screen was ordered. The blood cultures were negative. A blood smear was done to rule out malaria. Blood smears showed ring and trophozoites of *P. vivax* at 0.1% (Figure 1).

Malarial treatment with Artemether/Lumefantrine four tablets twice daily was initiated. Over the next 10 days, his symptoms resolved, and he was discharged from the designated COVID-19 facility. He received the Artemether/Lumefantrine for a total of 3 days, followed by 14 days of primaquine after ruling out G6PD deficiency, and was completely asymptomatic on 6 weeks follow-up in infectious disease clinic.

## Discussion

*Plasmodium vivax* is an intracellular parasite transmitted to humans by the bite of a female Anopheles mosquito. *P. vivax* malaria is a significant cause of morbidity in endemic areas. *P. vivax* can relapse by the activation of dormant liver-stage hypnozoites. Multiple relapses can follow a single mosquito inoculation.^6^ Infections such as *Plasmodium vivax* or *Plasmodium falciparum* malaria and certain bacterial infections are associated with relapse. However, there is no evidence of a malarial vivax relapse by viral illness^5^ Qatar is not an endemic area of *P. vivax*; however, imported malaria represents a significant threat to eliminating malaria in this region.^7^

The patient has a history of *P. vivax* infection, which was treated successfully a year ago, and the patient had been symptoms free since then. He had a history of exposure to COVID-19, after which he experienced fever, headache, and cough and was diagnosed as mild COVID-19 upper respiratory tract illness. Co-infections in COVID-19 are reported but not well-studied^8^; however, the persisting fever and malaria history raised suspicion of relapse due to COVID-19 infection. The patient received early treatment and made a complete clinical recovery.

The authors reviewed the literature on PubMed. Keywords used were (*Plasmodium vivax*) AND ((COVID-19) OR (SARS-CoV-2) OR (viral illness) OR (virus)). The search duration was from 1940 to October 7, 2020. 235 results were obtained and screened by two authors independently. 36 articles elaborating viral co-infections were found with dengue and HIV being the most common viral co-infections (Table 2). There has been a case report of COVID-19 and *Plasmodium vivax* malaria co-infection and another with possible reactivation of *P. vivax* secondary to SARS-CoV-2 co-infection.^9^ This is the second reported case of reactivation of *P. vivax* associated with COVID-19 and the first case of reactivation of imported malaria associated with COVID-19 in a non-endemic area. The similarity in the non-specific symptoms and febrile illness associated with COVID-19 and malaria makes missing a malaria diagnosis in the COVID-19 pandemic highly likely. Although the exact mechanism causing this activation is unclear, cytokine response associated with systemic illness has been postulated to induce vivax malaria relapses.^5^

**Table 2:** A Literature Review of *Plasmodium vivax* Co-Infection with Viruses

Number	Author	Type of Viral Co-Infection	PMID	Year of Publication
1.	Santana Vdos S et al	Dengue	21085859	2020
2.	Kishore, R et al	SARS-CoV-2	32621173	2020
3.	Sundus Sardar et al	SARS-CoV-2	32665888	2020
4.	Crystyan Siles et al	Guaroa virus	32186493	2020
5.	Luís A B Cruz et al	Hepatitis B virus (HBV)	31233500	2019
6.	Ana Cláudia Pereira Terças-Trettel et al	Hantavirus	31130600	2019
7.	Montenegro-Idrogo JJ et al	HIV	31800949	2019
8.	Wondimeneh Y. et al	HIV	30109850	2018
9.	Dewanee Ranaweera et al	HIV	30445967	2018
10.	Gebremeskel Tekle S et al	Varicella-Zoster	30014823	2018
11.	Vikarn Vishwajeet et al	Hepatitis B	29575054	2018
12.	Mohapatra PK et al	HIV	28749403	2017
13.	Tazeen A et al	Dengue and chikunguniya	28910810	2017
14.	Rao MR et al	Dengue	26653975	2016
15.	Vitor R R Mendonça et al	Dengue	26271921	2015
16.	Stępień M.	Dengue	26233086	2015
17.	Nicola Petrosillo et al	Ebola	26471197	2015
18.	Rattanapunya S et al	HIV	25728746	2015
19.	Magalhães BM et al	Dengue	25340346	2014
20.	Mushtaq, MB et al	Dengue	23606854	2013
21.	Magalhães BM et al	Dengue	23033396	2012
22.	Andrade, BB et al	Hepatitis B	21625634	2011
23.	Santana Vdos S et al	Dengue	21085859	2010
24.	McIver LJ et al	HIV	21413531	2010
25.	Chaudhry, R. et al	Leptospirosis dengue, Hepatitis E	19136807	2009
26.	Abbasi, A. et al	Dengue	19149976	2009
27.	Kaushik R.M. et al	Dengue	17568646	2007
28.	Braga WS et al	Hepatitis B	16501762	2006
29.	Deresinski S	Dengue	17283647	2006
30.	Thangaratham PS et al	Dengue	16785712	2006
31.	Braga WS et al	Hepatitis B	15895171	2005
32.	Bansal, R et al	Hepatitis E	12416764	2002
33.	Katongole-Mbidde E. et al	HIV	3130932	1998
34.	Hinrichsen SL. et al	HIV	8984995	1996
35.	Lo SS et al	HIV	1784955	1991
36.	Brown AE et al	Varicella-Zoster	1658946	1991

Authors believe that patients presenting with symptoms of fevers, headaches, and myalgias should be investigated for malaria infection, especially if they belong to an endemic region or have a history of malaria. Early management can decrease morbidity and mortality.

## Conclusion

Co-infections in COVID-19 are reported but not well-studied.^9^ *Plasmodium vivax* relapse should be considered a potential differential diagnosis of febrile illness in any patient with a previous malaria vivax presenting with viral illness symptoms. Delays in recognition and appropriate treatment of malaria can increase morbidity and mortality.

## References

[1] Cheung KS, Hung IFN, Chan PPY, et al. Gastrointestinal manifestations of SARS-CoV-2 infection and virus load in fecal samples from a Hong Kong Cohort: systematic review and meta-analysis. *Gastroenterology*. 2020;159(1):81–95. doi:10.1053/j.gastro.2020.03.065 32251668 PMC7194936

[2] Montalvan V, Lee J, Bueso T, de Toledo J, Rivas K. Neurological manifestations of COVID-19 and other coronavirus infections: a systematic review. *Clin Neurol Neurosurg*. 2020;194:105921. doi:10.1016/j.clineuro.2020.105921 32422545 PMC7227498

[3] Zhai LL, Xiang F, Wang W, et al. Atypical presentations of coronavirus disease 2019 in a patient with acute obstructive suppurative cholangitis. *Clin Res Hepatol Gastroenterol*. 2020;44(6):e135–e140. doi:10.1016/j.clinre.2020.05.003 32482542 PMC7241319

[4] Singhania N, Bansal S, Singhania G. An atypical presentation of novel Coronavirus disease 2019 (COVID-19). *Am J Med*. 2020;133(7):e365–e366. doi:10.1016/j.amjmed.2020.03.026 32320693 PMC7167564

[5] White NJ. Determinants of relapse periodicity in Plasmodium vivax malaria. *Malar J*. 2011;10. doi:10.1186/1475-2875-10-297 PMC3228849 21989376

[6] Shanks GD, White NJ. The activation of vivax malaria hypnozoites by infectious diseases. *Lancet Infect Dis*. 2013;13(10):900–906. doi:10.1016/S1473-3099(13)70095-1 23809889

[7] Farag E, Bansal D, Chehab MAH, et al. Epidemiology of malaria in the state of Qatar, 2008–2015. *Mediterr J Hematol Infect Dis*. 2018;10(1):e2018050. doi:10.4084/mjhid.2018.050 30210743 PMC6131099

[8] Yousaf Z, Khan AA, Chaudhary HA, et al. Cavitary pulmonary tuberculosis with COVID-19 coinfection. *IDCases*. 2020;22:e00973. doi:10.1016/j.idcr.2020.e00973 33014710 PMC7521360

[9] Sardar S, Sharma R, Alyamani TYM, Aboukamar M. COVID-19 and Plasmodium vivax malaria co-infection. *IDCases*. 2020;21:e00879. doi:10.1016/j.idcr.2020.e00879 32665888 PMC7305490

